# Evaluation of the Prevalence of Mucous Retention Pseudocyst and its Correlation with the Associated Risk Factors Using Panoramic Radiography and Cone-Beam Computed Tomography

**Published:** 2018-03

**Authors:** Mahdi Niknami, Mahdi Mirmohammadi, Azade Pezeshki

**Affiliations:** 1 Assistant Professor, Department of Oral and Maxillofacial Radiology, School of Dentistry, Tehran University of Medical Sciences, Tehran, Iran; 2 Dentist, Private Practice, Tehran, Iran

**Keywords:** Mucocele, Panoramic Radiography, Cone-Beam Computed Tomography, Maxillary Sinus

## Abstract

**Objectives:**

Mucous retention pseudocyst (MRP) of the maxillary sinus is an incidental finding on radiographs. The radiographs taken for dental purposes provide an opportunity for dentists to recognize asymptomatic maxillary sinus anomalies. The purpose of this study was to determine the prevalence of MRP on panoramic and cone-beam computed tomography (CBCT) views and to evaluate the associated risk factors.

**Materials and Methods:**

In this study, 710 panoramic radiographs and 90 CBCT scans were examined with regard to the presence of MRP in the maxillary sinus during 2014–15. The MRP prevalence and some associated risk factors such as age, gender, season, smoking, allergy, asthma, chronic sinusitis, nasal polyp, mucosal thickening, and post-nasal drip (PND) were evaluated.

**Results:**

The frequency of MRP was 2.4% on the 710 evaluated panoramic images and 43.3% on the 90 evaluated CBCT views. The frequency of MRP on the panoramic and CBCT views was higher in males than in females. There was a significant association between smoking and MRP on panoramic images (P=0.02) and CBCT views (P<0.001). There was a significant association between PND and MRP on CBCT views (P=0.02). The highest frequency of MRP was seen in spring (P=0.04) according to panoramic radiographs and in spring and summer (P=0.001) according to CBCT views.

**Conclusions:**

The occurrence of MRP had a significant association with smoking and PND, and the highest frequency of MRP was detected in spring and summer. Also, CBCT scanning detects MRP more accurately than panoramic radiography.

## INTRODUCTION

Mucous retention pseudocyst (MRP) is a benign and self-limiting lesion resulting from the outflow of mucus within the sinus mucosa due to ductal obstruction [1]. It is suggested that MRP is of non-odontogenic origin since it may occur in both dentate and edentulous patients [1]. Nevertheless, it is emphasized that periapical and periodontal diseases associated with maxillary molars, allergic reactions, trauma, smoking, and alteration of air temperature and humidity may be important etiological factors for sinusitis and MRP [2]. As the pathogenesis of MRP seems to be based on hypothesis, many names have been attributed to this lesion including pseudocyst, the retention cyst of the maxillary sinus, serous cyst, mucous cyst, and benign maxillary mucous cyst. A pseudocyst has no epithelial lining and is surrounded by a fibrous connective tissue [3–7].

**Fig. 1:**
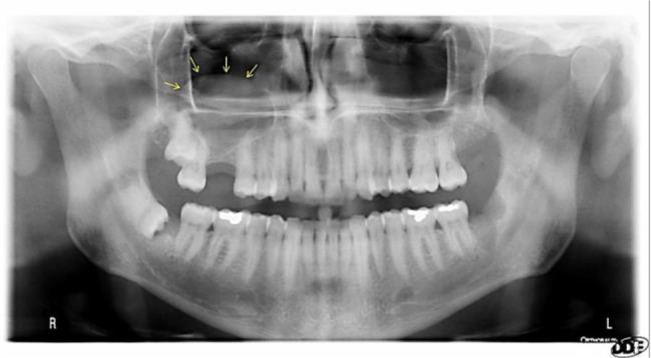
Mucous retention pseudocyst (MRP) of the right maxillary sinus on panoramic view

**Fig. 2:**
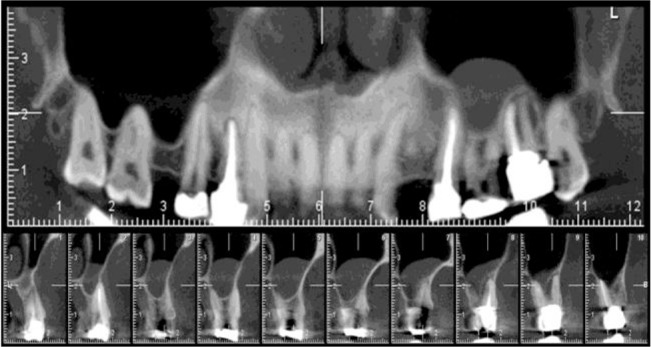
Mucous retention pseudocyst (MRP) of the left maxillary sinus on cross-sectional and panoramic-like CBCT views

The MRP is commonly found during radiographic examinations performed for other reasons. The lesion appears on panoramic and periapical radiographs of posterior maxillary teeth as a well-defined, homogeneous, dome-shaped, hemispherical or circular radiopacity of different dimensions, most commonly located on the floor of the maxillary sinus while preserving the sinus walls (Fig. 1). When the MRP completely fills the maxillary sinus, the radiographic interpretation becomes difficult since its appearance may mimic maxillary sinusitis [1–8]. Cone-beam computed tomography (CBCT), a recent technological development, provides three-dimensional images of mineralized maxillofacial tissues with negligible distortion and with radiation doses significantly lower than that of medical CT [9–13]. CBCT can be considered an important tool for the diagnosis of maxillary sinus alterations and for treatment planning [7–14] (Fig. 2). Few studies have compared panoramic radiography and CBCT in the detection of the changes in the maxillary sinus [11].

Although conventional radiographic images provide a two-dimensional representation of a three-dimensional structure, they are the primary available diagnostic tools and they represent a noninvasive method for the diagnosis of maxillary complications and for treatment planning [8]. Panoramic radiography has been shown to be a proper modality for epidemiological studies, and its imaging technique makes it well suited for the evaluation of the floor and posterior wall of the maxillary sinus [15–19]. The aim of the current study was to determine the prevalence and the associated risk factors of MRP on panoramic and CBCT views in different seasons.

## MATERIALS AND METHODS

In this cross-sectional study, CBCT scans and panoramic radiographs of the patients referring to the dental clinic of Tehran University of Medical Sciences during 2014–15 were assessed with regard to the presence of MRP. All the protocols for this experiment were approved by the Ethics Committee of Tehran University of Medical Sciences (code: 8811272015). In this study, 710 panoramic radiographs and 90 CBCT scans were independently evaluated. The images of the patients in the mixed dentition phase or with a history of rhinoplasty or sinus surgery and the images with technical errors were excluded. All the patients were informed about the nature of the study, and the demographic data including age, gender, medical history, seasonal allergy, smoking habits, evidence of asthma, chronic sinusitis, post-nasal drip (PND), nasal polyp, and history of sinus surgery were collected. The panoramic views were prepared by using Orthopantomograph® OP200 D (Instrumentarium Dental, PaloDEx Group Oy, Tuusula, Finland) with the exposure settings of 66 kilovoltage peak (kVp), 9.4 milliamperes (mA), and 14.1 seconds. The CBCT scans were obtained by using Alphard Vega CBCT system (Asahi Roentgen Ind. Co., Ltd., Kyoto, Japan) with the exposure settings of 80 kVp, 4 mA, and 17 seconds. The images were evaluated simultaneously by two oral and maxillofacial radiologists with regard to the presence of MRP and mucosal thickening of the maxillary sinus. A multiple logistic regression was used to investigate the relationship between MRP and the associated risk factors. P-values less than 0.05 were considered significant.

## RESULTS

The prevalence and the associated risk factors of MRP on panoramic and CBCT views are presented in Tables 1 to 7. The frequency of this lesion was 3% among males and 2% in females on panoramic images. The logistic regression tests showed that the odds ratio (OR) in males is 1.51 in comparison to females; however, the difference was not significant (P>0.05). The frequency of MRP on CBCT images was 52.9% in males and 37.5% in females (OR=1.88, Table 1). According to the findings on the panoramic images, the frequency of this lesion in 16-30-year olds was higher than that in the other two age groups; however, the difference was not significant (P>0.05). On CBCT images, MRP was observed in 52.6% of the patients under the age of 30 years (OR=3.21). Also, MRP was seen in 55.6% (OR=3.61) and 25.7% (OR=1) of the patients between the ages of 31–50 years and over the age of 50 years, respectively (Table 2).

**Table 1.** The frequency of mucous retention pseudocyst (MRP) according to gender

**Imaging technique**	**Gender**	**Number (percentage) of samples**	**Right side N(%)**	**Left side N(%)**	**Bilateral N(%)**	**Total N(%)**
Panoramic	Male (n=305)	296 (97)	2 (0.7)	3 (1)	4 (1.3)	9 (3)
	Female (n=405)	397 (98)	4 (1)	3 (0.7)	1 (0.2)	8 (2)

CBCT	Male (n=34)	18 (52.9)	6 (17.6)	4 (11.8)	8 (23.5)	16 (47.1)
	Female (n=56)	21 (37.5)	6 (10.7)	9 (16.1)	6 (10.7)	35 (62.5)

CBCT=Cone-Beam Computed Tomography

**Table 2.** The frequency of mucous retention pseudocyst (MRP) according to age

**Imaging technique**	**Age**	**MRP N(%)**	**OR**	**95% CI**	**P-value**
Panoramic	<30 years (n=230)	6 (2.6)	1.27	0.36–4.59	0.71
	31–50 years (n=285)	7 (2.5)	1.20	0.35–4.16	0.77
	>50 years (n=195)	4 (2.1)	1	-	-

CBCT	<30 years (n=19)	10 (52.6)	3.21	0.99–10.42	0.05
	31–50 years (n=36)	20 (55.6)	3.61	1.32–9.85	0.01
	>50 years (n=35)	9 (25.7)	1	-	-

OR=Odds Ratio, CI=Confidence Interval, CBCT=Cone-Beam Computed Tomography

**Table 3.** The frequency of mucous retention pseudocyst (MRP) according to medical history

**Imaging technique**	**Medical history**		**MRP N(%)**	**OR**	**95% CI**	**P-value**
Panoramic	Chronic sinusitis	Yes (n=23)	1 (4.3)	1.91	0.24–15.02	0.54
		No (n=687)	16 (2.3)	-	-	-
	Polyp	Yes (n=23)	1 (4.3)	1.91	0.24–15.02	0.54
		No (n=687)	16 (2.3)	-	-	-
	PND	Yes (n=87)	1 (1.1)	0.44	0.06–3.37	0.43
		No (n=687)	16 (2.6)	-	-	-

CBCT	Chronic sinusitis	Yes (n=10)	7 (70)	3.50	0.84–14. 55	0.08
		No (n=80)	32 (40)	-	-	-
	PND	Yes (n=21)	14 (66.7)	3.25	1.26–9.88	0.02
		No (n=69)	25 (36.2)	-	-	-

OR=Odds Ratio, CI=Confidence Interval, CBCT=Cone-Beam Computed Tomography, PND=Post-Nasal Drip

**Table 4.** The frequency of mucous retention pseudocyst (MRP) according to seasonal allergy

**Imaging technique**	**Attendance of Allergy**		**MRP N(%)**
Panoramic	Allergy	Yes (n=88)	2 (2.3)
		No (n=622)	15 (2.4)
	Allergy and season of imaging	Yes (n=37)	1 (2.7)
		No (n=51)	1 (2)

CBCT	Allergy	Yes (n=23)	13 (56.5)
		No (n=67)	26 (38.8)
	Allergy and season of imaging	Yes (n=10)	6 (60)
		No (n=13)	7 (53.8)

CBCT=Cone-Beam Computed Tomography

**Table 5.** The frequency of mucous retention pseudocyst (MRP) according to mucosal thickening

**Imaging technique**	**Mucosal thickening**	**MRP N(%)**			

		**No**	**Right side**	**Left side**	**Bilateral**
Panoramic	No	649 (97.9)	5 (0.8)	4 (0.6)	5 (0.8)
	Right side	11 (84.6)	-	2 (15.4)	-
	Left side	17 (100)	-	-	-
	Bilateral	16 (94.1)	1 (5.9)	-	-

CBCT	No	35 (70)	5 (10)	7 (14)	3 (6)
	Right side	7 (53.8)	-	3 (23.1)	3 (23.1)
	Left side	3 (33.3)	6 (66.7)	-	-
	Bilateral	6 (33.3)	3 (16.7)	3 (16.7)	6 (33.3)

CBCT=Cone-Beam Computed Tomography

**Table 6.** The frequency of mucous retention pseudocyst (MRP) according to smoking habits

**Imaging technique**	**Smoking**	**MRP N(%)**
**Panoramic**	Yes (n=54)	4 (7.4)
	No (n=656)	13 (2)

**CBCT**	Yes (n=20)	17 (85)
	No (n=70)	22 (31.4)

CBCT=Cone-Beam Computed Tomography

**Table 7.** The frequency of mucous retention pseudocyst (MRP) according to season

**Imaging technique**	**Season**	**MRP N(%)**
Panoramic	Spring (n=177)	8 (4.5)
	Summer (n=190)	-
	Autumn (n=165)	4 (2.4)
	Winter (n=178)	5 (2.8)

CBCT	Spring (n=23)	14 (60.9)
	Summer (n=10)	8 (80)
	Autumn (n=35)	8 (22.9)
	Winter (n=22)	9 (40.9)

CBCT=Cone-Beam Computed Tomography

The frequency of MRP on panoramic and CBCT images according to the medical history is presented in Table 3. According to the data obtained from the panoramic images, there was no significant correlation between the incidence of MRP and a history of chronic sinusitis, polyp, asthma, sinus surgery, or PND (P>0.05). On the CBCT images, MRP was seen in 70% of the patients with chronic sinusitis (P=0.08) and in 14 cases with PND (P=0.02). There was no significant correlation between the existence of MRP on CBCT images and a history of polyp, asthma, or sinus surgery (P>0.99). As observed in Table 4, MRP was detected on panoramic images of only 2.3% of the patients with a seasonal allergy. Additionally, there was a correlation between allergy and season of radiographic examination and the presence of MRP in 2.7% of the patients (OR=0.94). MRP was detected on CBCT images of 56.5% of the patients with an allergy. Also, there was a correlation between allergy and season of CBCT examination in 60% of the patients (OR=2.37).

The MRP frequency on the panoramic images according to the presence of mucosal thickening is presented in Table 5. As observed, mucosal thickening was detected in 47 (6.62%) patients.

No significant correlation was detected between the frequency of MRP on CBCT images and the presence of mucosal thickening (P>0.99). According to the results, MRP was detected on panoramic images of 7.4% of the smokers (4 cases), while it was detected on CBCT scans of 85% of the smokers (OR=12.36, Table 6). As shown in Table 7, on the panoramic images, MRP was detected more frequently in spring (4.5%) than in summer (0.0%). On the CBCT images, MRP was significantly more prevalent in spring and summer (OR=4.71) compared to other seasons.

## DISCUSSIN

Radiographic examinations provide the chance for dentists to investigate the changes in maxillary sinuses [1]. MRP is an asymptomatic condition that is detected incidentally on common radiographs such as panoramic and CBCT images [2,20, 21]. These cysts can expand and occupy the entire sinus cavity and can burst by sudden pressure changes caused by sneezing or exhalation. When the cyst shows a significant enlargement, it may become symptomatic and it may require treatments [1,16].

The etiology of the lesion is not clear and may be related to allergic or inflammatory processes, trauma, periapical and periodontal infections, humidity, or temperature. MRP can appear in any of the sinuses and at any time of the year, and based on previous studies, it is more prevalent during early spring and fall [1,4–7].The purpose of this study was to investigate the prevalence of MRP and the effect of some associated risk factors by using panoramic and CBCT imaging. We evaluated a wider range of risk factors in comparison with previous studies.Based on a study by Rodrigues et al [12], the accuracy of CBCT imaging outweighs that of panoramic radiography in detecting MRP. According to the results of our study, the prevalence of MRP on panoramic radiographs was 2.4%, whereas Rodrigues et al [12] reported a prevalence of 3.19%, and Casamassimo and Lilly [4] mentioned the prevalence of 1.6%. Furthermore, this prevalence was reported to be 1.5% in a study by ImaniMoghaddam et al [15], 7% in a study by Abesi et al [14], and 4% in a survey by Nemati et al [13].

The difference among the results could be due to the varying numbers of samples and different geographic and weather conditions. According to the results of the current study, no significant correlation was found between different age groups and the incidence of MRP on panoramic images. Casamassimo and Lilly [4] reported the highest rate of MRP in the third decade of life, whereas ImaniMoghaddam et al [15] and Nemati et al [13] reported a higher rate of MRP in the third and fifth decades. However, no significant correlation between age and the MRP prevalence was stated in the cited studies [4,13, 15].

In our study, the prevalence of MRP on panoramic images was 3% in males and 2% in females. ImaniMoghaddam et al [15] found this prevalence to be 4.8% in males and 5.4% in females. In the study by Nemati et al [13], the prevalence of MRP was calculated to be 6.2% in males and 2.3% in females. Abesi et al [14] declared the prevalence of 10.8% in males and 4.2% in females. In all the previous studies, except for the study by ImaniMoghaddam et al [15], the prevalence of MRP in males outweighed the prevalence in females [13,14].

In the current study, no significant correlation was detected between the MRP prevalence and gender, which agrees with the findings of ImaniMoghaddam et al [15]. However, in the studies by Abesi et al [14] and Nemati et al [13], a significant correlation was found between gender and the incidence of this lesion.

In the present study, similar to the study by Nemati et al [13], the numbers of the pseudocysts found in the right and left maxillary sinuses on panoramic images were equivalent. In the study by Casamassimo and Lilly [4], the right maxillary sinus was reported to be more frequently involved than the left sinus (55% vs 45%). Likewise, ImaniMoghaddam et al [15] reported the prevalence of the lesion to be higher in the right maxillary sinus (55.6% in the right sinus and 36.1% in the left sinus).

In the current study, 29.4% of the cases were bilateral. In the survey by ImaniMoghaddam et al [15], 8.3% of the cases were bilateral, while Nemati et al [13] found bilateral pseudocysts in 18.7% of the cases. It seems that there is no considerable difference between the left and right maxillary sinuses in terms of the frequency of the lesion, and most cases are unilateral [13,15].

The results of panoramic radiographic examinations showed that MRP is more prevalent in spring. In the survey by Nemati et al [13], the effect of different seasons on the incidence of this lesion was found to be significant as the lesion was seen more frequently in spring followed by fall. However, according to the study by Casamassimo and Lilly [4], the frequency of MRP was higher in September (late summer). Abesi et al [14] did not find any significant correlation between the MRP prevalence and different months of the year. Also, in the survey performed by Rodrigues et al [12], there was no significant correlation between the MRP prevalence and different months of the year, humidity, or temperature [12]. The results achieved by the evaluation of the panoramic radiographs showed that smoking considerably increases the prevalence of MRP, which agrees with the results found by Abesi et al [14]. However, ImaniMoghaddam et al [15] did not find a correlation between smoking and the MRP incidence. No significant relationship was found between seasonal allergies and MRP in the present study. However, Casamassimo and Lilly [4], ImaniMoghaddam et al [15], and Abesi et al [14] found a significant correlation between seasonal allergies and the MRP incidence. We did not find any significant correlation between the incidence of MRP and chronic sinusitis, nasal polyps, asthma, PND, or mucosal thickening.

In the present study, CBCT was utilized in addition to panoramic radiography, whereas only panoramic radiography has been used for investigating the MRP incidence and the probable risk factors in other studies [4,12–15]. The prevalence of MRP on the obtained CBCT images was 43.3%, which is significantly higher than the incidence found on the panoramic radiographs (2.4%).

This difference can be due to different sample volumes, to the ability of producing different sections in CBCT imaging, and to the investigation of different sections of the sinus without superimposition of other anatomic regions.

Similar to the panoramic images, the prevalence of MRP on the CBCT images was higher in males, and it was observed to be unilateral.

On both CBCT and panoramic images, a significant correlation was observed between the MRP prevalence and the seasons of the year as the prevalence of MRP was higher in spring and summer. Also, on both CBCT and panoramic images, a significant correlation was found between smoking and the presence of MRP.

Contrary to the panoramic images, a significant correlation was observed between the MRP incidence on CBCT views and PND. The incidence of mucosal thickening of the maxillary sinus on the CBCT images was 44.4%, which was considerably higher than the incidence on the panoramic views (6.62%).

However, in both imaging techniques, no correlation existed between the incidence of MRP and mucosal thickening of the maxillary sinus. On both CBCT and panoramic images, no relationship existed between MRP and other investigated risk factors including chronic sinusitis, nasal polyps, asthma, and a history of maxillary sinus surgery.

## CONCLUSION

The incidence of MRP showed a significant association with smoking and PND, and the highest frequency of MRP was detected in spring and summer. The evaluation of mucosal thickening of the maxillary sinus showed that CBCT is considerably more accurate than panoramic imaging in the detection of MRP. According to the results, it is recommended to use CBCT with a larger sample size in future studies.
